# Supplementary material for: ‘Degraded’ RNA profiles in Arthropoda and beyond
Source: PeerJ. 2015 Dec 1;3:e1436. doi: 10.7717/peerj.1436 (PMC4671170; doi:10.7717/peerj.1436)
Supplement: Figure S1 — Species aligned were Scolopendra subspinipes (HQ402538.1), Lepas anatifera Dosima fascicularis (KF781345.1) and Pollicipes pollicipes (EU370441.1). Shading in black signifies bases conserved across two or more species. Percent (%) IdentityMatrix—created by Clustal2.1. is also shown. [file peerj-03-1436-s001.pdf]

```
Scolopendra    1 GTCTA----AC-------------------------------------------------
Lepas          1 GTTTTAAAAACATTCGACCTCGGGGGTGCTGGACTACGCACTGAACTTAAGCATATCATT
Dosima         1 AATG--------------------------------------------------------
Pollicipes     1 GGC---------------------------------------------------------

Scolopendra    8 --------------------------------AT---GTGCGCGAGTCG-CAGGGTTGA-
Lepas         61 AAGTGCAGGAAAAGAAACCAAATGGGATTCCCATAGTAACGGCGAGTGAAGTGGGACGAG
Dosima         5 ------------------------GGATTCCCATAGTAACGGCGAGTGAAGTGGGATGAG
Pollicipes     4 ------------------------------------------------------------

Scolopendra   31 CTCGCAAGTTGAAACCCGAAGGCGAAATG--AAAGTGAAAGTCGGTGCGTCTGGCCGACT
Lepas        121 CTCAGC-ACCGAAGCCGGCGGTCGAAAGGCCGTCGTGCAATGTGGTGTTC----------
Dosima        41 CTCAGC-ACCGAAGCCGGCGGTCGAGAGGCCGTCGTGCAATGTGGTGTTT----------
Pollicipes     4 ------------------------------CGTCGTGCAATGTGGTGTTC----------

Scolopendra   89 GAGGCGGGATCCGTCCG-----------------GGTATCGCTGTAGCCCTTGAG-GGGG
Lepas        170 -GGGAGGGCTCTGTCCGTGTCCGGTGTTGGTCCAAGTCTCCTG---------GAAAGGAG
Dosima        90 -GGGAGGGCTCTGTCCGTGTCCGGTGTTGGTCCAAGTCTCCTG---------GAAAGGAG
Pollicipes    24 -GGGAGGGCCATGTCCGTGGCCGGCGTCGGTCCAAGTCTCCTG---------GAAAGGAG

Scolopendra  131 CTGCG---------GGA-GCCC-GGCGGCGCACCGCC-----------------------
Lepas        220 TTCCGTGGAGGGTGACAGGCCCGTGCGACCAATTGCCGCGTTCGCGGACGAGTCCTTCTT
Dosima       140 TTCCGTGGAGGGTGACAGGCCCGTGCGACCAATTGCCGTGCTCGCGGACGAGTCCTCCTT
Pollicipes    74 TTCCGTGGAGGGTGACAGGCCCGTGCGACCGATAGCCGCGTTCGCGGACGAGTCCTCCTT

Scolopendra  157 ------------------------------------------------------------
Lepas        280 CGAGTCGGGTTGTTCGCTAATGCAGCCCTAAGCAGGTGGTAAGCTCCATCTAAAGCTAAA
Dosima       200 CGAGTCGGGTTGTTCGCTAATGCAGCCCTAAGCAGGTGGTAAGCTCCATCTAAAGCTAAA
Pollicipes   134 CGAGTCGGGTTGTTCGCTAATGCAGCCCTAAGCAGGTGGTAAGCTCCATCTAAAGCTAAA

Scolopendra  157 ------------------------------------------------------------
Lepas        340 TATGACCACGAGTCCGATAGTCAACAAGTACAGTGATGGAAAGTTGAAAAGTACTCTGAA
Dosima       260 TATGACCACGAGTCCGATAGTCAACAAGTACAGTGATGGAAAGTTGAAAAGTACTCTGAA
Pollicipes   194 TACAACCACGAGTCCGATAGTCGACAAGTACAGTGATGGAAAGTTGAAAAGTACTCTGAA

Scolopendra  157 ------------------------------------------------------------
Lepas        400 GAGAGAGTCAAATAGCCCGTGAAGCTGCCAGTATGGGGTTAAGCGCACGTGTGACGCGTG
Dosima       320 GAGAGAGTCAAATAGCCCGTGAAGCTGCCAGTATGGGATTAAGCGCACGTGTGACGCGTG
Pollicipes   254 GAGAGAGTCAAATAGCCCGTGAAGCTGCCAGTATGGGATTAAGCGCACGTGTGACGCGTG

Scolopendra  157 ------------------------------------------------------------
Lepas        460 TCGTCGGACAAATAGGCGACTGCCGCA-G-CGTGCGGTCGGCGCGGCTG-GC-GGTGCGC
Dosima       380 TCGTTGGACAAATAGGCGACTGCCGGT-G-CGTGCGGTCGGCGCGGCGC-GCTTCT--GC
Pollicipes   314 TCGTCGGACAAATAGACGACCGTCGGCTGTCGCGCGATG--TTCGGCGCCAC-TCTTCGC

Scolopendra  157 ------------------------------------------------------------
Lepas        516 AAGCATCGCGT---CGCGTCGGCGCCGGCGCGCTGGACAGAACGCTTCCCTTTGTTCGAC
Dosima       435 GCG--TCGCGTCGATGCNNNNNNNNNNNNNNNNNNNNNNNNNNNNNNNNNNNNNNNNNNN
Pollicipes   371 GGG--TGGTGCCG-TGCGTCGTTAGCCGGGCGGCCGGCGGAACGTTTCCCTTTGTTCGGC

Scolopendra  157 ------------------------------------------------------------
Lepas        573 ACTGACAGGTCGGGGCTTCGTTGTGCGGTCTTATTGCCGACAGTGATCTGCAGAGCCGTG
Dosima       493 NNNNNN-N-NNNNNNNNNNNNNNNNNNNNNNNNNNNNNNNNNNNNNNNNNNNNNNNNNNN
Pollicipes   428 ATCGGCAGGTCGGGGCTTCGTTGTCCGGTCTTATTGCCGGTCGCCATCTGCAGAGCCGTG
```

```
Scolopendra  157 ------------------------------------------------------------
Lepas        633 CTGGGCGGTCGCCGTTTTCGGACGG-TGGCCGTGTTCCGTTGACTGTGG-CT-CTTCGCG
Dosima       551 NNNNNNNNNNNNNNN-NNNNNNNNNNNNNNNNNNNNNNNNNNNNNNNNNNNNNNNNNNNN
Pollicipes   488 TCGGGCCGGCGCCTCCTTCGGGTGTGTGCCGGTGCTCCGTGGACTGTGCGCGCTTCCATG

Scolopendra  157 ------------------------------------------------------------
Lepas        690 AGCG-CAACGGCCGGCTTGGTCTGCGGTGCAATG---CTGTCGT---GTGCGAACGACTG
Dosima       610 NNNN-NNNNNNNNNNNNNNNNNNNNNNNNNNNNN---NNNNNNNNNNNNNNNNNNNNNNN
Pollicipes   548 CGCGGCCACGGCCGGCCTGGTCTGCGGTGTGATGTGACCGCCAT---GTGCAAACGACTG

Scolopendra  157 ------------------------------------------------------------
Lepas        743 TGCGACGGCCAACGC-ATTCGA---GCTTTGCTCGGCTCGGCCTTTGCGTCGTCACGTGC
Dosima       666 NNNNNNNNNNNNNNN-NNNNNN---NNNNNAATCGGCTCGGCCTTTGCGTCGTCACGTGC
Pollicipes   605 GGCGACGGCCAGCGCAAATCGACTCGCAAGAGTCGGCTCGGCCTTTGCGTCGTCACGTGC

Scolopendra  157 GGCCCGTCTCGA--------CC------------------------GCTT----------
Lepas        799 GGCCCGTCTTGTAACACGGACCAAGGAGTCTAACATGTGAGCGAGCGATTGAGATTGACA
Dosima       722 GGCCCGTCTTGAAACACGGACCAAGGAGTCTAACATGTGAGCGAGCGATTGAGATTGACA
Pollicipes   665 GGCCCGTCTTGAAACACGGACCAAGGAGTCTAACATGTGAGCGAGCGATTGAGATTGTCG

Scolopendra  175 ---C-----------------------------CTGTCGGC-------------------
Lepas        859 AATCTCAAACGCGCAATGAAAGTGAAGTGTCGTCTGACGGCACGAGCGGTGATCCG--GC
Dosima       782 AATCTCAAACGCGCAATGAAAGTGAAGTGTCGTCTGACGGCACGAGCGGTGATCCG--GC
Pollicipes   725 AATCTCAAACGCGCAATGAAAGTGAAGTGTCGTCTGACGGCACGAGCGGAGATCCGCGGC

Scolopendra  184 ----------------------------G-------------------------------
Lepas        917 GTCACGTGTGGCGC--CGGCGCAACCGCGGGCCGCGCCATGGGCTGGCTTGCTTTTCGGA
Dosima       840 CTCA--AA----AC--CGGCGCAACCGCGGGCCGCGCCATGGGCTGGCTTGCTTTTCGGA
Pollicipes   785 GTGT--TATCGCGCCGCGGCGCATCCGCGGGCCGCGCCATTGGCTGGCTTGCTTTTCGGA

Scolopendra  185 ---------AG--GCGGAGCAAGAGCGCACACGTTGGGACCCGAAAGATGGTGAACTATG
Lepas        975 GCACTGCTCAGGCGCGGACCTAGAGCCCACACGTTGGGACCCGAAAGATGGTGAACTATG
Dosima       892 GCACTGCTCAGGCGCGGACCTAGAGCCCACACGTTGGGACCCGAAAGATGGTGAACTATG
Pollicipes   843 GCACTGCTAAGGCGCGGACCTAGAGCCCACACGTTGGGACCCGAAAGATGGTGAACTATG

Scolopendra  234 CTCGGGCAGGACGAAGCCAGAGGAAACTCTGGTGGAGGTCCGTAGCGATTCTGACGTGCA
Lepas       1035 CCTGGCCAGGATGAAGCCAGAGGAAACTCTGGTGGAGGTCCGCCGCGATTCTGACGTGCA
Dosima       952 CCTGGCCAGGATGAAGCCAGAGGAAACTCTGGTGGAGGTCCGCCGCGATTCTGACGTGCA
Pollicipes   903 CCTGGCCAGGATGAAGCCAGAGGAAACTCTGGTGGAGGTCCGCCGCGATTCTGACGTGCA

Scolopendra  294 AATCGATCGTCGGACCTGAGTATAGGGGCGAAAGACTAATCGAACCGTCTAGTAGCTGGT
Lepas       1095 AATCGATCGTCTGAGCTGGGTATAGGGGCGAAAGACCAATCGAACCATCTAGTAGCTGGT
Dosima      1012 AATCGATCGTCTGAGCTGGGTATAGGGGCGAAAGACCAATCGAACCATCTAGTAGCTGGT
Pollicipes   963 AATCGATCGTCTGAGCTGGGTATAGGGGCGAAAGACCAATCGAACAATCTAGTAGCTGGT

Scolopendra  354 TCCCTCCGAAGTTTCCCTCAGGATAGCTGGTGCTCGGTGAGGAACGCAGTCTCATCCGGT
Lepas       1155 TCCGCCCGAAGTTTCCCTCAGGATAGCTGGCGCTT-GTGTGATACGGAGTTTCATCCGGT
Dosima      1072 TCCGCCCGAAGTTTCCCTCAGGATAGCTGGCGCTT-GTGTGATACGGAGTTTCATCCGGT
Pollicipes  1023 TCCGCCCGAAGTTTCCCTCAGGATAGCTGGCGCTC-GTGTGATACGGAGTTTCATCCGGT

Scolopendra  414 AAAGCGAATGATTAGAGGCCTTGGGGCCGAATCGACCTCAACCTATTCTCAAACTTTCAA
Lepas       1214 AAAGCGAATGATCAGAGGAGCTGGGCTCCTTGCGAGCTCAACCTATTCTCAAACTTTCAA
Dosima      1131 AAAGCGAATGATCAGAGGAGCTGGGCTCCTTGCGAGCTCAACCTATTCTCAAACTTTCAA
Pollicipes  1082 AAAGCGAATGATCAGAGGAGCTGGGCTCCTTGCGAGCTCAACCTATTCTCAAACTTTCAA
```

```
Scolopendra  474 TGGGTGAGAAGTCCGGCTTGCCTGACTGAAGCCCGGACCAAATCTGATCCGACAGGGTGT
Lepas       1274 TGGGTGAGACGTCTCGCTGGCTTCAGTGCAGCGAC-------------------------
Dosima      1191 TGGGTGAGACGTCTCGCTGGCTTCAGTGCAGCGAC-------------------------
Pollicipes  1142 TGGGTGAGACGTCTCGCTGGCTTCACTGCAGCGAC-------------------------

Scolopendra  534 CGATCTGACAGTGTTGTCGGGGAGTAAAATCCTCGGCAACCTGGCTGCGGCCCTGCTCGG
Lepas       1309 ----------------------------------------------------------GA
Dosima      1226 ----------------------------------------------------------GA
Pollicipes  1177 ----------------------------------------------------------GA

Scolopendra  594 TA-CGGATGCGAGTGCCCAGTGGGCCAATTTTGGTAAGCAGAACTGGCGCTGTGGGATGA
Lepas       1311 CTCTGAATCCGAGTGCCCAGTGGGCCACTTTTGGTAAGCAGAACTGGCGATGCGGGATGA
Dosima      1228 CTCTGAATCCGAGTGCCCAGTGGGCCACTTTTGGTAAGCAGAACTGGCGATGCGGGATGA
Pollicipes  1179 CTCTGAATCCGAGTGCCCAGTGGGCCACTTTTGGTAAGCAGAACTGGCGATGCGGGATGA

Scolopendra  653 ACCAAACG-CCGGGTTACGGCGCCCGATGCGGACGCTCATCAGACCCCATGAAAGGTGTT
Lepas       1371 ACCGCATG-TCGAGTTAAGGTGCCT-AAGCGAACGCAAATCAGATACCATGAAAGGTGTT
Dosima      1288 ACCGCATG-TCGAGTTAAGGTGCCT-AAGCGGACGCAAATCAGATACCATGAAAGGTGTT
Pollicipes  1239 ACCGCATGGTCGAGTTAAGGTGCCT-AAGCGGACGCAAATCAGATACCATGAAAGGTGTT

Scolopendra  712 GGTTGCTATAGACAGCAGGACGGTGGCCATGGAAGTCGGAATCCGCTAAGGAGTGTGTAA
Lepas       1429 GATTGCTCACGACAGCAGGACGGTGGCCATGGAAGTCGGCATCCGCTAAGGAGTGTGTAA
Dosima      1346 GATTGCTCACGACAGCAGGACGGTGGCCATGGAAGTCGGCATCCGCTAAGGAGTGTGTAA
Pollicipes  1298 GATTGCTCACGACAGCAGGACGGTGGCCATGGAAGTCGGCATCCGCTAAGGAGTGTGTAA

Scolopendra  772 CAACTCACCTGCCGAAGCAACTAGCCCTGAAAATGGATGGCGCTCCAGCGTCGAGCCGAT
Lepas       1489 CAACTCACCTGCCGAAGCAATCAGCCCTGAAAATGGATGGCGCTAAAGCGTTCCACCGAT
Dosima      1406 CAACTCACCTGCCGAAGCAATCAGCCCTGAAAATGGATGGCGCTAAAGCGTTCCACCGAT
Pollicipes  1358 CAACTCACCTGCCGAAGCAATCAGCCCTGAAAATGGATGGCGCTAAAGCGTCCCACCGAT

Scolopendra  832 ACCCGGCCGCCGG-GGCAGAGACTTTGTGGCGA--TCCTACAAAAGAAATGCCCCGGTGA
Lepas       1549 ACTCGACCGTCGTCGGCAT-----GCGAGGCGCCAT-GTGCGCTTCTGACGCCACGACGA
Dosima      1466 ACTCGACCGTCGTCGGCAT-----GCGATGCGCCAT-GCGCGCATCTGACGCCACGACGA
Pollicipes  1418 ACTCGACCGTCGTCGGCAT-----GCGATGCTT-----TACGCATCTGACGCCACGACGA

Scolopendra  889 GTAGGAGGGTCGCGGCGGTGTGCGTCGAAGGTGTCGGGCGTAGGCCCGCCTGGAGCCACC
Lepas       1603 GTAGGGCGG-CGCGGCGGTGAGCGTTGAAGGCGTA-ACCGTGAGGTTAGCTGGAGCCGCC
Dosima      1520 GTAGGGCGG-CGCGGCGGTGAGCGTTGAAGGCGTA-ACCGTGAGGTTAGCTGGAGCCGCC
Pollicipes  1468 GTAGGGCGG-CGCAGTGGTGAGCGTTGAAGGCGTA-GCCGTGAGGCTAGCTGGAGCCGCC
                 *****  ** *** * **** **** ***** **    ***  *     ******** **

Scolopendra  949 GCCGGTGCAGATCTTGGTGGTAGTAGCAAATACTCGAGTGAGAACCTTGAGGACTGAAGT
Lepas       1661 GTCGGTGCAGATCTTGGTGGTAGTAGCAAATACACAAGCAAGATCCTTGTGGACCGATGT
Dosima      1578 GTCGGTGCAGATCTTGGTGGTAGTAGCAAATACACAAGCAAGATCCTTGTGGACCGATGT
Pollicipes  1526 ATTGGTGCAGATCTTGGTGGTAGTAGCAAATACACAAGCAAGATCCTTGTGGACCGATGT
                    ****************************** *  *  *** ***** **** ** **

Scolopendra 1009 GGAGAAGGGTTCCATGTGAACAGCAGTTGAACATGGGTCAGTCGGCCCTAAGG-------
Lepas       1721 GGAGAAGGGTTCCGCGCGAACAGTATTTGGACGTGGGTGAGTCGGCCCTAAGCCAACGGG
Dosima      1638 GGAGAAGGGTTCCGCGCGAACAGTATTTGGACGTGGGTGAGTCGGCCCTAAGCCAACGGG
Pollicipes  1586 GGAGAAGGGTTCCGCGCGAACAGTACTTGGACGTGGGTGAGTCGGCCCTAAGCCAACGGG
                 *************  * ******   *** ** ***** *************

Scolopendra 1062 ------------------------------------------------------------
Lepas       1781 TAATTCCCGCATCCCGATGTTTCTA-TCAATACA-AACGAAATACAACGGGTTGATGGCG
Dosima      1698 TAATTCCCGCATCC-------------CAA------------------------------
Pollicipes  1646 TAATTCCCGCATCCCG-TGTTACTGGCCAATATGTAAGCAAATAACATGGGTTGATGGCG
```

```
Scolopendra 1062 ------------------------------------------------------------
Lepas       1839 AAAGGGAATGCGGTCAATATTCCGCAACCAGACGCTGGCACAATGCTCGCTTCG-GCGAG
Dosima      1715 ------------------------------------------------------------
Pollicipes  1705 AAAGGGAATGCGGTCAATATTCCGCAACCGGACGCGGGCACAATGCTCACTTCGTGTGGG

Scolopendra 1062 ------------------------------------------------------------
Lepas       1898 CACGTCGCGGCGACGCTATCTGACTCGGTCTCGCTGATCATGACCCGAGGTAGAGTTTTC
Dosima      1715 ------------------------------------------------------------
Pollicipes  1765 CACGTCGCGGCGACGCCAACAGACTCGGTCTCGCTGACCATGACCCGAGGTAGAGTTTTC

Scolopendra 1062 ------------------------------------------------------------
Lepas       1958 TATTCTAATTGAGGGTTCCGGCCGTGGAAGCTATTCGCTATGAGAAACGAGAGTCTTTCG
Dosima      1715 ------------------------------------------------------------
Pollicipes  1825 TATTCTAGTTGAGGGTTCCGGCCGTGGAAGCTATTCGCTATGAGAAACGAGGGTCTTTCG

Scolopendra 1062 ------------------------------------------------------------
Lepas       2018 GGACTCGCGCATGGATACCCGCAGAGCGGTGTGTAATTCACGCCGTCTCGTGCGT-CATG
Dosima      1715 ------------------------------------------------------------
Pollicipes  1885 GGACTCGCGCATGGATACCCGCAGAGCGGTGTGTACTTCACGCCGTCTCGTGCGTCCATG

Scolopendra 1062 ------------------------------------------------------------
Lepas       2077 ATCGGCCCTTGAAAAACCGAGCGAGGGGACATGCTCAATGCCAGCGTCTGTCCGTACCCG
Dosima      1715 ------------------------------------------------------------
Pollicipes  1945 GTCGGCCCTTGAAACACCGAGCGAGGGGACATGCTCAATGCCCGCGTCCGTCCGTACCCG

Scolopendra 1062 ------------------------------------------------------------
Lepas       2137 ACACCGCATCAGGTCTCCAAGGTGAACAGCCTCTGGTCGATAGACGAATGTAGGTAAGGG
Dosima      1715 ------------------------------------------------------------
Pollicipes  2005 ACACCGCATCAGGTCTCCAAGGTGAACAGCCTCTGGTCGATAGACGAATGTAGGTAAGGG

Scolopendra 1062 ------------------------------------------------------------
Lepas       2197 AAGTCGGCAAAATGGATCCGTAACTTCGGGATAAGGATTGGCTCTGAGGCTTCGGTCGTT
Dosima      1715 ------------------------------------------------------------
Pollicipes  2065 AAGTCGGCAAAATGGATCCGTAACTTCGGGATAAGGATTGGCTCTGAGGCTTCGGTCGTT

Scolopendra 1062 ------------------------------------------------------------
Lepas       2257 CGGGCTCTTCTGTGAAGTGGTTAAGGTGCCGCGATCGCTGGGGCTAGGTCTTGTGCTGGT
Dosima      1715 ------------------------------------------------------------
Pollicipes  2125 CGGGCCTCTCTGTGAAGTGGTTAAGGTGCCGCGATCACTTGGGCTAGGTCTTGTGCTGGT

Scolopendra 1062 ------------------------------------------------------------
Lepas       2317 CACGAGACTTACTGGACCTTGTCCGCGTGTCATCCGCCATGGATCGGCTGAGCTTGTCGG
Dosima      1715 ------------------------------------------------------------
Pollicipes  2185 CACGAGACTTACTGGACCTTGTCCGCGTGCTACCCGCCATGGATCGGTTTGGCTTGTCGG

Scolopendra 1062 ------------------------------------------------------------
Lepas       2377 CCCGCTCGGCCGCC-GGGTGTTCGCC------TACACTT---C--------GGCGGTCGA
Dosima      1715 ------------------------------------------------------------
Pollicipes  2245 CCTGTCCGGCCGCGCGCGCTGTCGCGAGCGCTCGCGCGTGAGCGGCGCGCGCACGGTCGA

Scolopendra 1062 ------------------------------------------------------------
Lepas       2419 CGGGAACAACCGCGTCGTTCGGCAACTAAGAAGCAACTCAGAACTGGCACGGACCAGGGG
Dosima      1715 ------------------------------------------------------------
Pollicipes  2305 CGGGAACAACCGCGTCGTTCGGCAACTAAGAAGCAACTCAGAACTGGCACGGACCAGGGG
```

```
Scolopendra 1062 ------------------------------------------------------------
Lepas       2479 AATCCGACTGACTAATTAAATCATAGCATCGCGATGGCCAAGGTCGGTGTTGACGCGATG
Dosima      1715 ------------------------------------------------------------
Pollicipes  2365 AATCCGACTGACTAATTAAATCATAGCATCGCGATGGCCAAGGTCGGTGTTGACGCGATG

Scolopendra 1062 ------------------------------------------------------------
Lepas       2539 TGATTTCTGCCCAGTGCTCTGAATGTCAAAGTGAAGAAATTCATCCAAGCGCGGGTAAAC
Dosima      1715 ------------------------------------------------------------
Pollicipes  2425 TGATTTCTGCCCAGTGCTCTGAATGTCAAAGTGAAGAAATTCATCCAAGCGCGGGTAAAC

Scolopendra 1062 ------------------------------------------------------------
Lepas       2599 GGCGGGAGTAACTATGACTCTCTTAAGGTAGCCAAATGCCTCGTCATCTAATTAGTGACG
Dosima      1715 ------------------------------------------------------------
Pollicipes  2485 GGCGGGAGTAACTATGACTCTCTTAAGGTAGCCAAATGCCTCGTCATCTAATTAGTGACG

Scolopendra 1062 ------------------------------------------------------------
Lepas       2659 CGCATGAATGGATCAACGAGATTCCCACTGTCCCTATCTACTATCTAGCGAAACCACAGC
Dosima      1715 ------------------------------------------------------------
Pollicipes  2545 CGCATGAATGGATCAACGAGATTCCCACTGTCCCTATCTACTATCTAGCGAAACCACAGC

Scolopendra 1062 -----------------AAT----A--GGGGAA-----AC-CCGTTC-------------
Lepas       2719 CAAGGGAACGGGCTTGGATTCGTCAGCGGGGAAAGAAGACCCTGTTGAGCTTGACTCTAA
Dosima      1715 ------------------------------------------------------------
Pollicipes  2605 CAAGGGAACGGGCTTGGAATCGTCAGCGGGGAAAGAAGACCCTGTTGAGCTTGACTCTAA

Scolopendra 1080 --CGA--------AG-----CG--GGGTGT--T-----------------------CGTG
Lepas       2779 TCTGACTCTGTGAGGAGACATGGCAGGTGTAGGCTAGGTGGGAGGCGCAAGTCGACTGTG
Dosima      1715 ------------------------------------------------------------
Pollicipes  2665 TCTGACTCTGTGAGGAGACATGGCAGGTGTAGTCTAAGTGGGAGACGCAAGTCGAATGTG

Scolopendra 1098 CAACACGAC---CC----CGGCTGA-------C----CGA--AAGGGAATCGGGTTAA--
Lepas       2839 AAATACCACTACTTTCATTGTCTCATCACTAACACAGTGATAAGGGGACTCGATCACTTC
Dosima      1715 ------------------------------------------------------------
Pollicipes  2725 AAAGACCACTACTTTCATTGTCTCATCACTAACACAGTGATAAGGGGACTCGAACTCCTC

Scolopendra 1136 ----------TATTCCCGAACCCGGATGCGGAAGTC----------------GGACCCT-
Lepas       2899 G-GTGATCGCGATTCTCGATTCA--AGGCGTAAGGCGGCTTGTCCGTCTGGCCGACCCAC
Dosima      1715 ------------------------------------------------------------
Pollicipes  2785 GCGAGTTCGCGATTCTCGATTCA--AGGCGCAAGGCGGCTKGTCCGTCTGGCCGACCCAC

Scolopendra 1169 -------------TCG--------------------GGGTCCA------ATGCGGTAACG
Lepas       2956 GCTGAGGACACAGTCAGGTGGGGAGTTTGACTGGGGCGGTACATCTGTCAAACGATAACG
Dosima      1715 ------------------------------------------------------------
Pollicipes  2843 GCTGAGGACACAGTCAGGTGGGGAGTTTGACTGGGGCGGTACATCTGTCAAACGATAACG

Scolopendra 1190 CAAA----C------GAACTCGGAGACGTCG--------------GC-------------
Lepas       3016 CAGGTGTCCGAAGGCCAGCTCAGAGGCGACAGAAACGCCTCGTAGAGCAAAAGGGCAAAA
Dosima      1715 ------------------------------------------------------------
Pollicipes  2903 CAGGTGTCCGAAGGCCAGCTCAGAGGCGACAGAAACGCCTCGTAGAGCAAAAGGGCAAAA

Scolopendra 1213 ------------GGGAGTCCCGG-----------------GAAGAGTTGT---------C
Lepas       3076 GCTGGCTTGATCTGGATTTTCAGTACGAATACGGACCACGAAAGTGCGGCCCATCGATCC
Dosima      1715 ------------------------------------------------------------
Pollicipes  2963 GCTGGCTTGATCTGGATTTTCAGTACGAATACGGACCACGAAAGTGCGGCCCATCGATCC
```

```
Scolopendra 1235 TTTT-----------CTTT--GTAA-----------------------GGG---------
Lepas       3136 TTTTGATTTTACGAGC--------------------------------------------
Dosima      1715 ------------------------------------------------------------
Pollicipes  3023 TTTTGATTTTACGAGCTTTAAGCAAGAGGTGTCAGAAAAGTTACCACAGGGATAACTGGC

Scolopendra 1250 -----ACGG----------------AC-T-----------CCCTGG-AATCGGCTC----
Lepas       3152 ------------------------------------------------------------
Dosima      1715 ------------------------------------------------------------
Pollicipes  3083 TTGTGGCGGCCAAGCGTTCATAGCGACGTCGCTTTTTGATCCTTCGATGTCGGCTCTTCC

Scolopendra 1272 ------------------------------------------------------------
Lepas       3152 ---------------------TTT------------------------------------
Dosima      1715 ------------------------------------------------------------
Pollicipes  3143 TATCATTGYGAAGCAGAATTCRCCAAGYGTYGGATTGTTCACCCRCTAAYAGGGAACGTG

Scolopendra 1272 -----------GACCGG----AGAGAGGGATGT-------------TGTTCCCGT-----
Lepas       3155 ------------------------------------------------------------
Dosima      1715 ------------------------------------------------------------
Pollicipes  3203 AGCTGGGTTTAGACCGTCGTGAGACAGGTTAGTTTTACCCTACTGATGACCCCGTCTTCG

Scolopendra 1299 ---------------A---------AAGCACCGCGGC-TC------TTG---C-------
Lepas       3155 ------------------------------------------------------------
Dosima      1715 ------------------------------------------------------------
Pollicipes  3263 CCATGGCAATCCTGCTCAGTACGAGAGGAACCGCAGGTTCGGACATTTGGTACATGTGCT

Scolopendra 1318 -GG-TGT-------------------------CCG-------------GAGCGCTCCC--
Lepas       3155 ------------------------------------------------------------
Dosima      1715 ------------------------------------------------------------
Pollicipes  3323 CGGCTGACAAGCCGATGGTGCGAGGCTACCATCCGACGGATTATGACTGAACGCCTCTAA

Scolopendra 1336 -------GCCGGCCC------TTGAAAAT-CC--------------------GAGGGAGA
Lepas       3155 ------------------------------------------------------------
Dosima      1715 ------------------------------------------------------------
Pollicipes  3383 GTCAGAATCCGGCCCAGAAACGTGAAGATACCGTCTGTACCTCGACTGTCGGGAGGCAGA

Scolopendra 1362 GA------------ATT----------TG-------------ATTTTCG--CAC------
Lepas       3155 ------------------------------------------------------------
Dosima      1715 ------------------------------------------------------------
Pollicipes  3443 GATATGCGCGGCCAATTGCGGCCGCGGTGGGAAAAGCCACAGCTTTGCGGGCACGGCAAC

Scolopendra 1379 ---CGG-----------------------------------------ACCGTACC-----
Lepas       3155 ------------A-----------------------------------------------
Dosima      1715 -------TGTTTA-----------------------------------------------
Pollicipes  3503 TGCCGGGGGTCTAGGTAACCTCCTTGCCCTCGGCAGCCGAAGCCGAGATCGATCCCAGTT

Scolopendra 1390 --------------CATATC----CGCA-GCA
Lepas       3156 -----------A-------------GCA-AGA
Dosima      1721 -----------T-------------GC---AT
Pollicipes  3563 CAATGTAGAGGTGCCAAATCATTACGCAGACG
```

Percent (%) Identity Matrix - created by Clustal2.1

1: centipepde

2: pollicipes

3: lepas

4: dosima

	1	2	3	4
1:	100.00	69.11	68.95	73.64
2:	69.11	100.00	89.17	80.46
3:	68.95	89.17	100.00	83.45
4:	73.64	80.46	83.45	100.00
