# Supplementary material for: ‘Degraded’ RNA profiles in Arthropoda and beyond
Source: PeerJ. 2015 Dec 1;3:e1436. doi: 10.7717/peerj.1436 (PMC4671170; doi:10.7717/peerj.1436)
Supplement: Figure S2 — Samples lacking gap deletion display detectable RNA Integrity numbers (RINs) when heat denaturation is conducted prior to analysis. [file peerj-03-1436-s002.pdf]

Sample 10

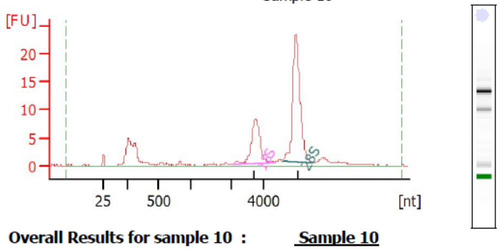

Overall Results for sample 10 : Sample 10

RNA Area:	135.6
RNA Concentration:	166 ng/µl
rRNA Ratio [28s / 18s]:	2.2
RNA Integrity Number (RIN):	9.2 (B.02.08)
Result Flagging Color:	
Result Flagging Label:	RIN: 9.20

Fragment table for sample 10 : Sample 10

Name	Start Size [nt]	End Size [nt]	Area	% of total Area
18S	2,085	4,655	25.3	18.6
28S	5,157	6,996	54.7	40.3

Sample 11

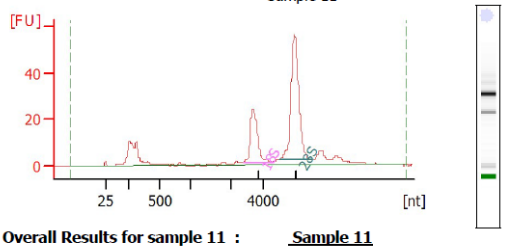

Overall Results for sample 11 : Sample 11

RNA Area:	353.7
RNA Concentration:	433 ng/µl
rRNA Ratio [28s / 18s]:	2.3
RNA Integrity Number (RIN):	9.4 (B.02.08)
Result Flagging Color:	
Result Flagging Label:	RIN: 9.40

Fragment table for sample 11 : Sample 11

Name	Start Size [nt]	End Size [nt]	Area	% of total Area
18S	2,786	4,613	59.7	16.9
28S	5,038	7,055	134.9	38.1

Sample 12

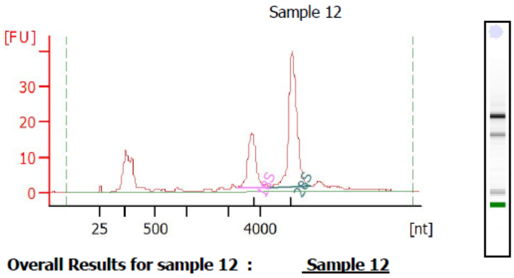

Overall Results for sample 12 : Sample 12

RNA Area:	282.4
RNA Concentration:	346 ng/µl
rRNA Ratio [28s / 18s]:	2.2
RNA Integrity Number (RIN):	9.2 (B.02.08)
Result Flagging Color:	
Result Flagging Label:	RIN: 9.20

Fragment table for sample 12 : Sample 12

Name	Start Size [nt]	End Size [nt]	Area	% of total Area
18S	2,527	4,591	44.8	15.9
28S	4,634	7,020	97.5	34.5
